# mRNA-Based Vaccine BNT162b2 Might Reduce Severe Acute Respiratory Syndrome Coronavirus 2 B.1.1.7 Variant Transmission in Japanese Population

**DOI:** 10.7759/cureus.19140

**Published:** 2021-10-30

**Authors:** Takuya Matsunaga, Yoshihito Higashidate, Natsuko Inazawa, Satomi Ando, Masahiro Takahashi

**Affiliations:** 1 Department of Medical Oncology, Japan Community Health Care Organization (JCHO) Sapporo Hokushin Hospital, Sapporo, JPN; 2 Department of Pediatrics, Japan Community Health Care Organization (JCHO) Sapporo Hokushin Hospital, Sapporo, JPN; 3 Department of Dermatology, Japan Community Health Care Organization (JCHO) Sapporo Hokushin Hospital, Sapporo, JPN; 4 Department of Surgery, Japan Community Health Care Organization (JCHO) Sapporo Hokushin Hospital, Sapporo, JPN

**Keywords:** japan, bnt162b2, mrna vaccine, sars-cov-2, b.1.1.7 variant

## Abstract

Coronavirus disease 2019 (COVID-19) cluster with severe acute respiratory syndrome coronavirus 2 (SARS-CoV-2) B.1.1.7 variant occurred between April 10, 2021, and May 26, 2021, at Japan Community Health Care Organization (JCHO) Sapporo Hokushin Hospital in Sapporo, Japan. We found that the four infected staff members accounted for 5.3% of all 75 infected persons, approximately one of 10 the percentage of other Japanese hospitals that experienced disease clusters caused by wild-type SARS-CoV-2 until January 2021. Furthermore, none of the infected staff developed COVID-19. Nationwide vaccination began in February 2021, when wild-type SARS-CoV-2 infection remained prevalent in Japan. During March-May, Sapporo had already experienced an explosive increase in SARS-CoV-2 B.1.1.7 cases. JCHO Sapporo Hokushin Hospital started optional vaccination for staff members using BNT162b2. The first inoculations occurred between February 22, 2021, and April 28, 2021, and the second between March 15, 2021, and May 7, 2021. This is the first report that BNT162b2 might reduce B.1.1.7 variant transmission in Japanese population.

## Introduction

Severe acute respiratory syndrome coronavirus 2 (SARS-CoV-2), the novel coronavirus that causes coronavirus disease 2019 (COVID-19), was first reported in Wuhan, China, in December, 2019. Since being declared a global pandemic by World Health Organization (WHO), COVID-19 damaged public healthcare systems and the disease cluster at hospitals frequently occurred in many countries. The pandemic also resulted in the loss of living due to prolonged closure, which had a ripple effect on the global economy. Even though substantial progress in clinical research has led to a better understanding of SARS-CoV-2 and the management of COVID-19, limiting the continuing spread of this virus has become an issue of increasing concern, as SARS-CoV-2 continues to wreak havoc across the world, with many countries enduring a third or fourth wave of outbreaks of this viral illness attributed mainly due to the emergence of mutant viruses. SARS-CoV-2 bearing B.1.1.7 lineage spike protein (B.1.1.7 variant), which emerged in the United Kingdom (UK) during December 2020, is more transmissible than wild-type SARS-CoV-2, and it may also increase COVID-19 severity and mortality [1,2]. The sera from individuals vaccinated with BNT162b2 had largely preserved neutralizing titers against the SARS-CoV-2-S pseudoviruses bearing B.1.1.7 spike protein [3,4]. However, whether BNT162b2 reduces SARS-CoV-2 B.1.1.7 variant transmission in humans had not been demonstrated. Recently, the effectiveness of BNT162b2 in preventing the B.1.1.7 variant infection in Italians and Israelis was reported [5-7]. However, whether BNT162b2 prevents B.1.1.7. variant infection in the Japanese population has not been reported yet.

## Materials and methods

Real-time reverse transcription-polymerase chain reaction (PCR) using saliva as a sample was performed to detect SARS-CoV-2. We used Ampdirect^TM^ 2019-nCoV detection kit (Shimadzu, Kyoto, Japan) and real-time PCR devise Cobas z 480 (Roche Diagnostics Co., Ltd., Tokyo, Japan) according to the manufacture’s protocol. The records of the COVID-19 cluster at Japan Community Health Care Organization (JCHO) Sapporo Hokushin Hospital (276 beds; disease cluster period: April 10, 2021, to May 26, 2021) were analyzed retrospectively. This study was reviewed and approved by the Ethics Committee of JCHO Sapporo Hokushin Hospital (No. 2021-3), and the need for informed consent was waived because there was no risk to the subjects. Our data were compared with those of four Japanese hospitals (Eiju General Hospital {400 beds; disease cluster period: March 20, 2020, to May 9, 2020}, Hokkaido Cancer Center {430 beds; disease cluster period: April 16, 2020, to June 13, 2020}, Juntendo University Nerima Hospital {490 beds; disease cluster period: September 28, 2020, to November 4, 2020}, and Asahikawa Kosei Hospital {539 beds; disease cluster period: November 22, 2020, to January 6, 2021}) that experienced disease clusters caused by wild-type SARS-CoV-2 until January 2021. The data were reproduced from their home pages. The antibody levels against SARS-CoV-2 S protein in our staff before and after vaccination were measured [8]. This experiment was reviewed and approved by the Ethics Committee of JCHO Sapporo Hokushin Hospital (No. 2021-2), and written informed consent was obtained. All procedures performed in studies involving human participants were in accordance with the ethical standards of the institutional and/or national committee and with the 1964 Helsinki declaration and its later amendments or comparable ethical standards.

## Results

**Figure 1:**
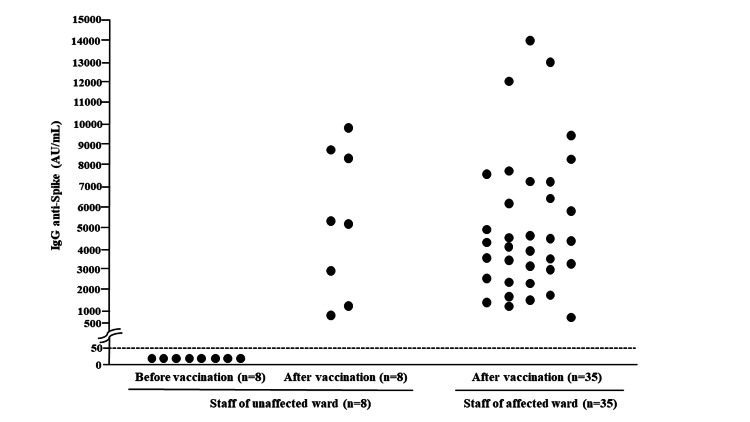
Significant increase in antibody titers against SARS-Cov-2 was obtained after vaccination in our staff. SARS-Cov-2: severe acute respiratory syndrome coronavirus 2

At JCHO Sapporo Hokushin Hospital in Sapporo, Japan, 17 inpatients, one doctor, and two nurses became infected with SARS-CoV-2 on April 10, 2021. The next day, another inpatient in the same ward also became infected. Afterward, all staff members and inpatients underwent PCR tests for SARS-CoV-2 every one to six days, depending on their risk. Patient zero must be in the affected ward from March 26-31, 2021, and developed COVID-19 on April 3, 2021. Therefore, discharged patients who coincided in the ward with this patient also underwent PCR tests. On April 21, 2021, another nurse in the affected ward tested positive. Between April 10 and April 21, 2021, four staff members, 41 inpatients, one dialysis outpatient, and 19 discharged patients became infected with SARS-CoV-2. Genome analysis performed by the Sapporo Institute of Public Health revealed B.1.1.7 in the disease cluster. During March-May, 2021, Sapporo had already experienced an explosive increase in SARS-CoV-2 B.1.1.7 cases. On May 26, 2021, we declared the end of the disease cluster. The final count of infected persons was 75 - four staff members, 51 inpatients, one dialysis outpatient, and 19 discharged patients. The four infected staff members accounted for 5.3% of all 75 infected persons, approximately one of 10 the percentage of four Japanese hospitals (Eiju General Hospital {43.2% of all 192 infected people}, Hokkaido Cancer Center {45.1% of all 82 infected people}, Juntendo University Nerima Hospital {60.0% of all 60 infected people}, and Asahikawa Kosei Hospital {41.8% of all 311 people}). None of the infected staff developed COVID-19. Nationwide vaccination began in February 2021, when wild-type SARS-CoV-2 infection remained prevalent in Japan. Our hospital started optional vaccination for all 429 staff members using BNT162b2. The first inoculations occurred during February 22 to April 28, 2021, and the second during March 15 to May 7, 2021. In total, 393 staff members received two vaccinations, five received one, and 31 received none. On April 2, 2021, 73.4% (315) of all staff were vaccinated once, and 69.2% (297) were inoculated twice. Of the infected staff, one received both shots (infected staff A), another received one (infected staff B), and the remaining two (infected staff C and D) were not vaccinated until April 2, 2021. We confirmed that a significant increase in antibody titers against SARS-CoV-2 was obtained after vaccination in our staff including four infected staff members (Figure 1).

The antibody titers against SARS-CoV-2 of infected staff A, infected staff B, infected staff C, and infected staff D were 6078 AU/mL, 3481 AU/mL, 2415 AU/mL, and 4151 AU/mL, respectively. We used a chemiluminescent microparticle immunoassay (SARS-CoV-2 IgG II Quant assay on an ARCHITECT analyzer {Abbott, Chicago, IL}) to quantify IgG antibodies in our staff's serum sample before and 12 weeks after the second vaccination. We could not obtain blood from the staff of the affected ward before vaccination. The assay detects antibodies against the receptor-binding domain of the S1 subunit of the spike protein of SARS-CoV-2. A value of ≥ 50 arbitrary units per milliliter (AU/mL) was considered evidence of vaccination response [8]. Although our staff had undetectable antibody levels before vaccination, defined as < 50 AU/mL, a significant increase in antibody titers against SARS-CoV-2 was observed after vaccination in our staff in both the unaffected and affected wards.

## Discussion

Our data suggest that BNT162b2 might reduce SARS-CoV-2 B.1.1.7 variant transmission in the Japanese population. In late December 2020, a new SARS-CoV-2 variant of concern, B.1.1.7 lineage was reported in the United Kingdom based on whole-genome sequencing of samples from patients who tested positive for SARS-CoV-2 [9,10]. It was reported to be more transmissible, surpassing preexisting variants of SARS-CoV-2 to emerge as the dominant SARS-CoV-2 variant in the United Kingdom [11]. In Japan, the first patient infected with SARS-CoV-2 B1.1.7 was found in the end of December 2020 and experienced an explosive increase in B.1.1.7 cases during March-May, 2021. A large matched cohort study performed in the United Kingdom reported that the mortality of the patients infected with B.1.1.7 lineage variant was higher than the patients with previously circulating wild-type strains [1]. Another study reported that the B.1.1.7 variant was associated with increased mortality compared to other SARS-CoV-2 variants [2]. The risk of death was reportedly greater among individuals with confirmed B.1.1.7 variant of concern compared with individuals with non-B.1.1.7 SARS-CoV-2 [12]. Besides the importance of imposing public health and infection control measures to prevent or decrease the transmission of SARS-CoV-2, the most crucial step to contain this global pandemic is vaccination to prevent SARS-CoV-2 infection in communities across the world. Extraordinary efforts by clinical researchers worldwide during this pandemic have resulted in the development of novel vaccines against SARS-CoV-2 at an unprecedented speed to contain this viral illness that has devastated communities worldwide. Vaccination triggers the immune system leading to the production of neutralizing antibodies against SARS-CoV-2. Results of multinational, placebo-controlled, observer-blinded, pivotal efficacy trial reported that individuals aged 16 years or older receiving two-dose regimen the trial vaccine BNT162b2 (mRNA-based, BioNTech/Pfizer) when given 21 days apart conferred 95% protection against COVID-19 with a safety profile similar to other viral vaccines [13]. Based on the results of this vaccine efficacy trial, the FDA issued an Emergency Use Authorization (EUA) on December 11, 2020, granting the use of the BNT162b2 vaccine to prevent COVID-19. Ministry of Health, Labour, and Welfare in Japan approved the use of the BNT162b2 vaccine to prevent COVID-19 on February 22, 2021. In vitro analysis of the serum samples obtained from 40 participants from the BNT162b2 clinical efficacy trial efficiently had slightly reduced but overall largely preserved neutralizing titers against the B.1.1.7 lineage pseudovirus [3]. The sera from individuals who received the vaccine exhibited a broad range of neutralizing titers against the wild-type pseudoviruses that were modestly reduced against the B.1.1.7 variant [4]. However, whether BNT162b2 reduces B.1.1.7 variant transmission in humans had not been clarified yet. Recently, the effectiveness of BNT162b2 in preventing the B.1.1.7 variant infection in Italians and Israelis was reported [5-7]. Sansone et al. demonstrated the effectiveness of the BNT162b2 vaccine against the B.1.1.7 variant of SARS-CoV-2 among healthcare workers (HCWs) in Brescia, Italy [5]. Vaccinated HCWs were at lower infection risk as compared to unvaccinated HCWs (by 2.6-folds), and even to a greater extent (6.2 folds) if compared to the general population. In the observation period, they observed 92 SARS-CoV-2 infections among HCWs, most among unvaccinated HCWs. Munittz et al. reported that (i) the B.1.1.7 variant is 45% more transmissible than the wild-type strain in Israel, (ii) active surveillance markedly reduces the transmission of B.1.1.7 in nursing homes, (iii) prioritized vaccination prevents B.1.1.7-associated infections in the elderly, (iv) proactive surveillance, combined with prioritized vaccination, is achievable [6]. Haas et al. showed that two doses of BNT162b2 are highly effective across all age groups in preventing symptomatic and asymptomatic SARS-CoV-2 infections and COVID-19-related hospitalization, severe disease, and death, including those caused by the B.1.1.7 SARS-CoV-2 variant [7].

Our study has two limitations. There might be other factors that influence the percentage of infected staff members per all infected persons in the outbreak situation other than vaccination. In the comparison of the JCHO Sapporo Hokushin Hospital outbreak with the outbreaks in the other hospitals that happened in the earlier period of the COVID-19 pandemic, there might be difference in the level of infection control measures and compliances to those measures in each hospital. Given that vaccination reduces asymptomatic infection with the B.1.1.7 SARS-CoV-2 variant,^ ^it is plausible that vaccination reduces transmission [7]. However, data from clinical trials are lacking. We provide empirical evidence suggesting that BNT162b2 might reduce SARS-CoV-2 B.1.1.7 variant transmission.

## Conclusions

This is the first report that BNT162b2 might reduce SARS-CoV-2 B.1.1.7 variant transmission in the Japanese population. We hope that a clinical trial will be conducted in Japan to investigate whether BNT162b2 reduces SRS-CoV-2 B.1.1.7 variant transmission. Furthermore, we strongly hope that vaccination will progress rapidly around the world, and the SARS-CoV-2 epidemic will end as soon as possible.

## References

[1] Challen R, Brooks-Pollock E, Read JM, Dyson L, Tsaneva-Atanasova K, Danon L (2021). Risk of mortality in patients infected with SARS-CoV-2 variant of concern 202012/1: matched cohort study. BMJ.

[2] Davies NG, Jarvis CI, Edmunds WJ, Jewell NP, Diaz-Ordaz K, Keogh RH (2021). Increased mortality in community-tested cases of SARS-CoV-2 lineage B.1.1.7. Nature.

[3] Muik A, Wallisch AK, Sänger B (2021). Neutralization of SARS-CoV-2 lineage B.1.1.7 pseudovirus by BNT162b2 vaccine-elicited human sera. Science.

[4] Collier DA, De Marco A, Ferreira IA (2021). Sensitivity of SARS-CoV-2 B.1.1.7 to mRNA vaccine-elicited antibodies. Nature.

[5] Sansone E, Tiraboschi M, Sala E, Albini E, Lombardo M, Castelli F, De Palma G (2021). Effectiveness of BNT162b2 vaccine against the B.1.1.7 variant of SARS-CoV-2 among healthcare workers in Brescia, Italy. J Infect.

[6] Munitz A, Yechezkel M, Dickstein Y, Yamin D, Gerlic M (2021). BNT162b2 vaccination effectively prevents the rapid rise of SARS-CoV-2 variant B.1.1.7 in high-risk populations in Israel. Cell Rep Med.

[7] Haas EJ, Angulo FJ, McLaughlin JM (2021). Impact and effectiveness of mRNA BNT162b2 vaccine against SARS-CoV-2 infections and COVID-19 cases, hospitalisations, and deaths following a nationwide vaccination campaign in Israel: an observational study using national surveillance data. Lancet.

[8] Grupper A, Sharon N, Finn T (2021). Humoral response to the Pfizer BNT162b2 vaccine in patients undergoing maintenance hemodialysis. Clin J Am Soc Nephrol.

[9] Volz E, Mishra S, Chand M (2021). Assessing transmissibility of SARS-CoV-2 lineage B.1.1.7 in England. Nature.

[10] Galloway SE, Paul P, MacCannell DR (2021). Emergence of SARS-CoV-2 B.1.1.7 lineage - United States, December 29, 2020-January 12, 2021. MMWR Morb Mortal Wkly Rep.

[11] Davies NG, Abbott S, Barnard RC (2021). Estimated transmissibility and impact of SARS-CoV-2 lineage B.1.1.7 in England. Science.

[12] Grint DJ, Wing K, Williamson E (2021). Case fatality risk of the SARS-CoV-2 variant of concern B.1.1.7 in England, 16 November to 5 February. Euro Surveill.

[13] Polack FP, Thomas SJ, Kitchin N (2020). Safety and efficacy of the BNT162b2 mRNA COVID-19 vaccine. N Engl J Med.

